# Supplementary material for: MiDAS 5: Global diversity of bacteria and archaea in anaerobic digesters
Source: Nat Commun. 2024 Jun 25;15:5361. doi: 10.1038/s41467-024-49641-y (PMC11199495; doi:10.1038/s41467-024-49641-y)
Supplement: Supplementary file 3 — Description of Additional Supplementary Files [file 41467_2024_49641_MOESM3_ESM.pdf]

Description of Additional Supplementary Files

File Name: Supplementary Data 1
Description: Metadata for the sampled anaerobic digesters.

File Name: Supplementary Data 2
Description: In silico determined primer coverage for all MiDAS taxa.

File Name: Supplementary Data 3
Description: Core and conditional rare and abundant taxa in anaerobic digesters.
